# Inflammation and Diabetic Kidney Disease

**DOI:** 10.3390/ijms27021097

**Published:** 2026-01-22

**Authors:** Rong Mei Zhang, Maria Luiza Caramori

**Affiliations:** 1 Department of Endocrinology and Metabolism, Medical Specialty Institute, Cleveland Clinic Foundation, Cleveland, OH 44106, USA; 2 Department of Heart, Blood and Kidney Research, Cleveland Clinic Research, Cleveland, OH 44106, USA; 3 Cleveland Clinic Lerner College of Medicine (CCLCM), Case Western Reserve University (CWRU) School of Medicine, Cleveland, OH 44106, USA; 4 Division of Endocrinology and Diabetes, Department of Medicine, University of Minnesota, Minneapolis, MN 55404, USA

**Keywords:** diabetic kidney disease, diabetes, inflammation, monocytes, chronic kidney disease

## Abstract

Diabetes is the leading cause of end-stage kidney disease and significantly contributes to morbidity and mortality in people with diabetes. Despite significant advances in the last decade, including the development of novel therapies, the residual risk in diabetic kidney disease (DKD) remains high. One yet unaddressed factor in the pathogenesis of DKD is immune activation. Early in DKD, there is infiltration of macrophages, T-cells, B-cells, and dendritic cells in mouse models, while at later stages, neutrophils are also observed. This review will highlight novel insights into the contribution of immune cells to the development of DKD, with a particular focus on the innate immune system and the cellular crosstalk between immune cells and intrinsic kidney cells as contributors to DKD. One example of this bidirectional crosstalk is observed between macrophages and podocytes. While macrophages can directly mediate podocyte injury and apoptosis via TNF-α secretion, podocytes secrete cytokines that further recruit macrophages. Understanding the role of immune-mediated injury in kidney disease is critical in reducing the residual risk of DKD.

## 1. Introduction

Diabetes is the leading cause of end-stage kidney disease, and affects up to 30% of people with type 2 diabetes (T2D) and 40% of people with type 1 diabetes (T1D) [1,2]. Diabetic kidney disease (DKD) is the leading cause of end-stage renal disease in the US and other developing countries [3]. Importantly, DKD is a significant contributor to the morbidity and mortality of people with diabetes, and has been found to increase all-cause mortality by 20–40 times compared to people without DKD [4]. DKD is the clinical diagnosis of chronic kidney disease (CKD) defined by an estimated glomerular filtration rate (eGFR) < 60 mL/min/1.73 m^2^ and/or the presence of albuminuria for ≥3 months in a person with diabetes [5]. Diabetic nephropathy (DN) refers to the presence of histopathological features of kidney injury caused by diabetes in a person’s kidney biopsy, including glomerular basement membrane thickening, mesangial expansion, nodular glomerulosclerosis, arteriosclerosis, and interstitial fibrosis [6]. However, histopathologic changes can occur in the absence of clinical manifestations, making early diagnosis, and thus early treatment, of DKD challenging.

Multiple factors contribute to the development of DKD including hyperglycemia, increased intraglomerular pressure, activation of the renin–angiotensin–aldosterone–system (RAAS), profibrotic factors, genetics, and immune activation [7]. Despite the use of current standard of care therapy targeting glycemia, blood pressure, and/or albuminuria with RAAS blockade, sodium-glucose cotransporter 2 inhibitors (SGLT2i), glucagon-like peptide-1 receptor agonists (GLP-1 RA), and non-steroidal mineralocorticoid receptor antagonists (MRA), the residual risk of DKD remains high [8]. A key unaddressed factor in the pathogenesis of DKD is immune activation, in particular activation of monocytes/macrophages, as a unifying factor underlying the pathogenesis of DKD. This occurs by hyperglycemia inducing release of cytokines and chemokines, which promotes recruitment of immune cells to the kidney, leading to fibrosis and intraglomerular hypertension [9]. This review will provide an update to studies relevant to the role of the immune system in DKD. Furthermore, we will bring new insight to the innate immune system and its cellular interactions in the kidney.

## 2. Innate Immunity

### 2.1. Monocytes/Macrophages

**Figure 1:**
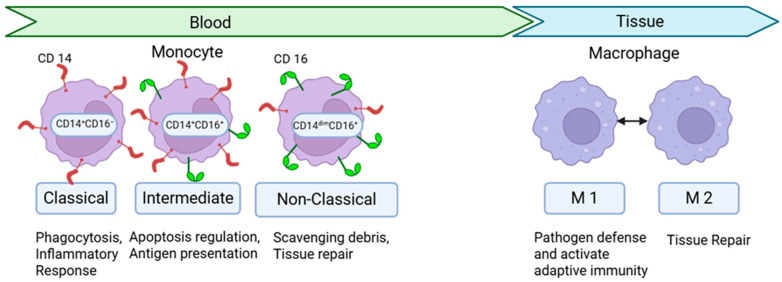
Human monocyte and macrophage subtypes Monocytes are recruited from the circulation to differentiate into macrophages in the tissue. Monocytes subtypes include classical (CD14^+^CD16^−^), intermediate (CD14^+^CD16^+^), and nonclassical (CD14^dim^CD16^+^). Classical monocytes play a role in phagocytosis, and in generating an inflammatory response. Intermediate monocytes play a role in apoptosis regulation, cytokine secretion, and antigen presentation. Nonclassical monocytes aid in the scavenging of debris, and in tissue repair and resolution of inflammation. Circulating monocytes can be recruited to the kidney to replenish tissue macrophages, where they are defined as M1 or M2 subtypes. M1 macrophages are proinflammatory and release cytokines to defend against pathogens and activate adaptive immunity. M2 macrophages are involved in tissue repair and release anti-inflammatory cytokines to control the immune response.

Monocytes make up 10% of circulating white blood cells, and are classified into several subtypes including classical (CD14^+^CD16^−^), intermediate (CD14^+^CD16^+^), and nonclassical (CD14^dim^CD16^+^) (Figure 1) [10]. Classical monocytes can be recruited from the circulation to the endothelium and tissues, where they can repopulate tissue macrophages. These macrophages can participate in phagocytosis, infection control, and the inflammatory response [10]. Alternatively, classical monocytes can also differentiate into dendritic cells. Nonclassical monocytes can remain in the circulation to take part in patrolling the vasculature, phagocytosis, and tissue repair/resolution of inflammation [10]. Intermediate monocytes are thought to play a role in antigen processing, cytokine secretion, and apoptosis regulation [10]. Additionally, there are resident macrophages in the kidney which are characterized by M1 or M2 subtypes, and exhibit roles in proinflammatory or resolving inflammation, respectively (Figure 1) [11]. Kidney resident macrophages can originate from the yolk sac, fetal liver, or hematopoietic stem cells [11]. These resident macrophages exhibit plasticity, and can switch between these phenotypes in response to environmental cues in the setting of DKD, as we will discuss in further detail later [11].

Recruitment of circulating monocytes to the kidneys is believed to be a key step in DKD. In the setting of hyperglycemia, intrinsic kidney cells such as podocytes, mesangial, and tubular cells are stimulated to secrete monocyte chemoattractant protein-1 (MCP-1), also known as C-C chemokine ligand 2 (CCL2), which binds to the C-C chemokine receptor 2 (CCR2) on monocytes and promote monocyte recruitment into the kidney [12]. Once in the kidneys, monocytes can bind to adhesion molecules such as intercellular adhesion molecular-1 (ICAM-1) or vascular cell adhesion molecule-1 (VCAM-1) to infiltrate the kidney, where monocytes can replenish resident macrophages and differentiate into either inflammatory or anti-inflammatory macrophage subtypes [12]. M1 macrophages are thought to play a role in the inflammatory injury phase of DKD, while M2 macrophages may play a role in the anti-inflammatory repair phase. An ineffective repair phase is believed to allow for persistent inflammation and fibrosis [9]. The importance of circulating monocytes in the development of kidney disease is seen in both animal models and humans with DKD.

Animal Studies

In a mouse model of T1D, hyperglycemia-induced macrophage infiltration in the kidney, glomerular hypertrophy, and albuminuria were attenuated by macrophage depletion, and this also prevented podocyte injury [13]. More recent single cell RNA sequencing (scRNA-seq) data confirmed the importance of macrophages in animal models of DKD. A T1D mouse model of early DKD showed increased numbers of infiltrating and resident macrophages in the kidneys of diabetic mice compared to controls. Over time, the M1 macrophage subtype population increased, while M2 macrophages decreased, demonstrating an increase in inflammatory activation over the course of DKD, and a potentially ineffective repair phase leading to kidney injury [14]. Similarly, these findings were also seen in a db/db mouse model of T2D, where there was higher macrophage accumulation in the glomerular and interstitial areas, which correlated with albuminuria, kidney function, and MCP-1 expression [15]. These data support the association between monocyte/macrophage infiltration and the presence of histopathological and clinical changes of DKD in animal models, and suggest that there might be a progressive shift toward a proinflammatory M1 macrophage phenotype as DKD progresses.

Human Studies

Monocytes/macrophages are also important contributors to human DKD. In a study of human kidney biopsies, there was significant accumulation of macrophages in the setting of moderate glomerulosclerosis in patients with classical DN lesions [16]. Another study observed higher macrophage accumulation in the interstitium of people with T2D compared to controls. In this study, macrophage accumulation in the interstitium was significantly correlated with the severity of kidney disease [17]. Recent studies suggest that the M1/M2 classification may oversimplify the functional heterogeneity of tissue macrophages in vivo. Tissue macrophages respond to local tissue cues that allow for activation and transcriptional shifts that allow them to develop disease-specific gene expression profiles and phenotypes [18]. This plasticity was observed in a recent scRNA-seq study of human biopsies of early DKD compared to those with minimal change disease, where increased anti-inflammatory TREM2^+^ and MRC1^+^ in addition to inflammatory S100A4^+^ macrophages were observed in the kidney [14]. These studies suggest that the presence of kidney monocytes/macrophages are associated with the development of DKD in humans. Further work is needed to delineate the heterogeneity of macrophage subtypes and their specific roles in DKD.

Another study found no difference in the number of macrophages per glomerulus in the kidney biopsies of people with T2D and DN compared to people with diabetes with no DN lesions and nondiabetic controls. However, they found a positive correlation between the number of glomerular anti-inflammatory CD163^+^ macrophages and both the histopathological classification of DN and the severity of interstitial fibrosis and tubular atrophy [19]. Despite this conflicting study showing a lack of increased macrophages in DN, the majority of studies do suggest an increase in the number of macrophages in DN. These studies suggest that, in humans, it is unclear if the presence of anti-inflammatory macrophages indicates ineffective repair leading to increased DN severity, while in animal models this may reflect a progressive cellular shift toward a proinflammatory phenotype. Cumulatively, these studies support an association between monocytes/macrophages and the severity of DN, but the contribution of macrophage subtypes to the progression of DKD needs to be clarified.

#### 2.1.1. Cellular Interactions

##### Monocytes/Macrophages-Podocytes

Animal Studies

**Figure 2:**
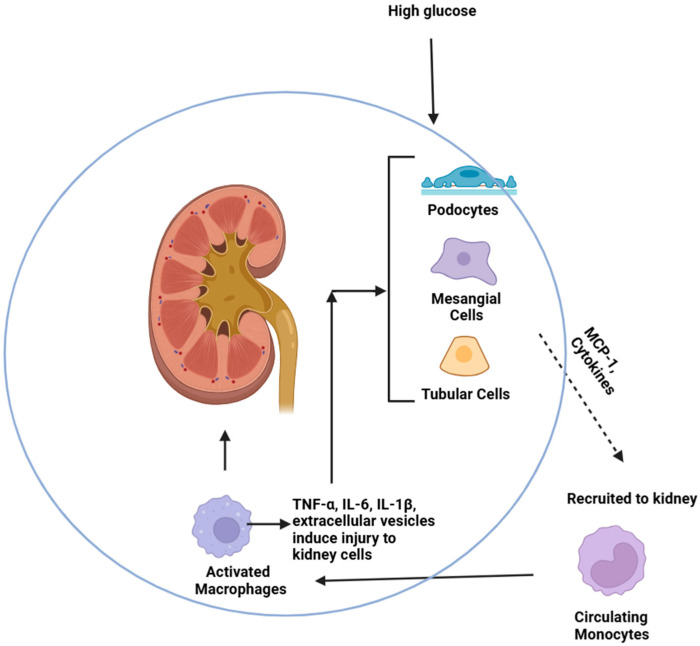
Recruitment of monocytes to the kidney mediates intrinsic injury. Kidney cells, including podocytes, mesangial, and tubular cells, secrete chemokines such as MCP-1 in response to high glucose, which bind to CCR2 to recruit circulating monocytes to the kidney. Once in the kidney, monocytes differentiate into macrophages to replenish resident macrophages and secrete cytokines and extracellular vesicles to mediate injury to intrinsic kidney cells. Macrophages exposed to high glucose secrete TNF-α, which induces podocyte apoptosis or tubular necroptosis. Macrophages exposed to high glucose can also secrete exosomes, which are internalized by mesangial cells to promote cytokine secretion.

Upon recruitment into the kidney, monocytes and macrophages can exert direct effects on intrinsic kidney cells. Several animal studies indicate direct cellular interactions between monocytes/macrophages and podocytes. MCP-1 is a chemokine secreted by podocytes to attract monocytes to the kidney (Figure 2). In an in vitro study, murine macrophages exposed to high glucose exhibited increased migration toward podocytes compared to cells exposed to normal glucose, and this was attributed to podocyte secretion of MCP-1 [13]. Furthermore, this study found that inflammatory M1 macrophages induced increased podocyte permeability compared to M2 macrophages [13]. This study highlights how high glucose directly induces podocyte recruitment of macrophages, which in turn promotes podocyte injury (Table 1). Several podocytesignaling pathways have been implicated in monocyte/macrophage-induced podocyte injury, one of which was the tumor necrosis factor-α (TNF-α) pathway. In an in vitro model, RAW 247 murine macrophages, a mouse macrophage line, were shown to secrete TNF-α in response to high glucose, which directly induced podocyte apoptosis by activating reactive oxygen species (ROS) in a p38 map kinase-dependent manner [20]. Neutralizing TNF-α effectively attenuated podocyte apoptosis by reducing ROS and p38 map kinase activation in this model, to suggest TNF-α is important in inducing podocyte injury [20].

Additional mediators of podocyte injury in the monocyte secretome are extracellular vesicles. An in vitro study demonstrated that high glucose induced extracellular vesicle secretion from murine macrophages, leading to increased ROS and inflammasome activation in podocytes [21]. These data support the concept of a bidirectional macrophage–podocyte crosstalk contributing to DKD.

Human Studies

Our recent work also found that exposure of monocytes from healthy individuals to high glucose in vitro for 6 h induced increased TNF-α secretion [22]. Of note, in this study, in vitro TNF-α secretion was inversely correlated with serum levels of vitamin D in these healthy individuals [22]. Exposure of human podocytes to conditioned high glucose monocyte media increased podocyte apoptosis compared to exposure to normal glucose monocyte media [22]. These studies show the importance of TNF-α as a direct mediator of podocyte injury in both human and mouse models. It remains to be determined whether podocyte apoptosis also occurs in a ROS-p38 map kinase-dependent mechanism in humans as it appears to in animal models.

##### Monocytes/Macrophages–Glomerular Endothelial Cells

Animal Studies

Macrophages also directly interact with glomerular endothelial cells (Table 1). In a streptozocin (STZ)-induced diabetic mouse model of T1D and DKD, there was higher M1 macrophage accumulation in the glomerulus prior to the onset of glomerular senescence [23]. Conversely, glomerular endothelial cells activated in the setting of diabetes can also directly recruit macrophages to the glomerulus. In a db/db mouse model of T2D, activated glomerular endothelial cells exhibited endothelial dysfunction, which increased recruitment of M1 macrophages, and was prevented by treatment with a peroxisome proliferator-activated receptor-α agonist [24].

Human Studies

The cellular crosstalk between macrophages and glomerular endothelial cells was also seen in human models. In vitro studies showed that co-culture of THP-1 M1 macrophages, a human monocytic cell line, with human glomerular endothelial cells induced higher senescence in the glomerular endothelial cells by activating ROS and p38 map kinase [23]. In both human and animal models, M1 macrophage accumulation appears important to induce glomerular endothelial dysfunction or senescence in the development of DKD. Cumulatively, these studies support direct cellular interactions between monocyte/macrophages and glomerular cells, which lead to cellular injury by activating aberrant signaling and cytokine secretion for direct cellular injury.

##### Monocytes/Macrophages–Mesangial Cells

Animal Studies

Macrophages also interacted directly with mesangial cells in the glomerulus based on studies in animal models. Murine macrophages exposed to high glucose in vitro exhibited a M1 phenotype, which induced the release of exosomes. These exosomes were internalized by mesangial cells, which led to a significant increase in TNF-α, interleukin 6 (IL-6), and IL-1β release, and inhibited autophagy [25]. The importance of exosomes as a mediator of cellular crosstalk was further supported by a murine in vitro study, which showed high glucose promoted macrophage exosome secretion, which was taken up by mesangial cells to trigger cytokine secretion and extracellular matrix formation [26]. Despite these studies indicating a role for macrophage-derived exosomes in triggering mesangial cytokine secretion, this mechanism has only been demonstrated in animal models, and future studies are needed to define whether it also occurs in humans with DKD.

##### Monocytes/Macrophages–Tubular Cells

Animal Studies

Though the traditional pathogenesis highlights glomerular injury as an important mechanism in DKD, emerging evidence indicates tubular injury, characterized by tubular hypertrophy and mononuclear cell infiltration, may predate the onset of albuminuria [27]. As in macrophage-induced podocyte injury, TNF-α is also an important mediator of tubular injury. In a recent study, RAW 264.7 murine macrophages, a mouse macrophage cell line, treated to high versus normal glucose in vitro had increased tubular epithelial cell necroptosis via increased TNF-α secretion, due to activation of the NOTCH signaling pathway in macrophages [28]. Tubular cells also contribute to macrophage recruitment. In db/db mice, hyperglycemia-induced IL-1β secretion from tubular cells, which promoted an inflammatory macrophage phenotype and enhanced macrophage infiltration to the kidney [29]. These studies highlight a feedback loop between tubular cells recruiting monocytes/macrophages, which leads to tubular injury. As a common mechanism in animal models of DKD demonstrated so far in podocytes and tubular cells, hyperglycemia stimulates macrophage recruitment to the kidney by stimulating chemokine/cytokine secretion from intrinsic kidney cells. Once in the kidney, macrophages mediate podocyte or tubular cell injury via TNF-α secretion, underscoring the importance of TNF-α as a mediator of injury in DKD.

Human Studies

In people with DKD, an important pattern recognition receptor in the recruitment of monocytes to the kidney tubules is toll-like receptor 4 (TLR4). In vitro studies confirmed exposure to high glucose induced TLR4 protein expression in human tubular cells, and silencing of TLR4 expression by small interfering RNA reduced mononuclear cell migration by reducing IL-6 and chemokine ligand 2 expression [30]. People with DN showed higher TLR4 protein expression in the kidney tubules, which correlated with the degree of interstitial macrophage infiltration, and showed an inverse correlation with eGFR [30]. These studies confirm robust crosstalk between macrophages and tubular cells in animal and human models.

**Table 1:** Summary of monocyte/macrophage interactions with kidney cells.

**Monocytes/Macrophages-Podocytes**	
	Animal Studies - Murine macrophages had higher migration to podocytes in high glucose due to podocyte secretion of MCP-1 [13]. - Murine macrophages induced higher podocyte apoptosis due to TNF-α [20]. - High glucose induced extracellular vesicle secretion from murine macrophages to cause reactive oxygen species and inflammasome activation in podocytes [21]. Human Studies - Monocytes from people exposed to high glucose in vitro induced higher podocyte apoptosis compared to normal glucose [22].
**Monocytes/Macrophages-Glomerular Endothelial Cells**	
	Animal Studies - A streptozocin-induced diabetic mouse model had higher M1 macrophage accumulation in the glomerulus [23]. - Glomerular endothelial cells had endothelial dysfunction, which increased recruitment of M1 macrophages [24]. Human Studies - Macrophages cocultured with human glomerular endothelial cells (HGEC) in vitro induced higher senescence in HGEC [23].
**Monocytes/Macrophages-Mesangial Cells**	
	Animal Studies - Macrophages exposed to high glucose in vitro induced exosome release, which were internalized by mesangial cells and led to cytokine release [25,26].
**Monocytes/Macrophages-Tubular Cells**	
	Animal Studies - High glucose induced TNF-α secretion from macrophages to induce tubular cell necroptosis [28]. - In db/db mice, hyperglycemia induced IL-1β secretion from tubular cells to enhance macrophage infiltration to the kidney [29]. Human Studies - High glucose in vitro promoted TLR4 expression in human tubular cells. Silencing TLR4 reduced mononuclear cell migration [30].

### 2.2. Neutrophils

Neutrophils are the most abundant cells of the immune system and are critical for the innate immune system’s rapid response to eradicating pathogens. Neutrophils have proinflammatory granules, which contain neutrophil gelatinase-associated lipocalin (NGAL) [31]. Upon activation, neutrophils eliminate pathogens through several mechanisms including generation of ROS, secretion of proinflammatory enzymes, and release of neutrophil extracellular traps (NETs) into the surrounding space.

Animal Studies

In animal models, in vitro exposure of mouse glomerular endothelial cells to NETs induced higher NOD-like receptor family, pyrin domain containing 3 inflammasome activation, leading to IL-1β secretion and endothelial dysfunction, which were attenuated with NET inhibition [32]. In a diabetic mouse model, exposure to hyperglycemia induced increased expression of NET-related proteins in the kidneys of mice that also exhibited tubulointerstitial inflammation [33]. However, in this model, NETs were not seen in early stages of kidney disease, suggesting that they may be associated with later disease stages. Further evidence for the pathogenic role of NETs is seen in a mouse model of DKD, where immunostaining for NETs showed a higher number of NETs in the glomerulus compared to controls, which correlated with albuminuria and fractional mesangial area [32]. Use of a NET formation inhibitor in mice attenuated NET in the glomerulus, and led to a reduction in albuminuria [32]. These animal model studies suggest that neutrophils may play a role in later stages of DKD by promoting both glomerular and tubulointerstitial injury. While macrophages mediate injury by cytokines/chemokines release as discussed above, neutrophils can induce damage to intrinsic kidney cells by release of NETs.

Human Studies

Evidence for the role of neutrophils in DKD is limited in humans. In human biopsy studies, there was increased infiltration of macrophages, neutrophils, and B-cells in the tubulointerstitial area, and higher NET accumulation in the glomeruli of people with DKD compared to people without diabetes [34].

### 2.3. Dendritic Cells

Dendritic cells are antigen-presenting cells which activate T-cells by presenting antigens on major histocompatibility complexes [35]. Dendritic cells can be derived from myeloid, lymphoid, or monocyte precursors. They can be activated by stimuli including hyperglycemia and advanced glycation end-products. In healthy individuals, dendritic cells are present in the kidney, and remain immature to allow for tolerance to self-antigens from T-cells [35]. However, in response to acute stimuli, dendritic cells activate T-cells and macrophages to generate an immune response.

Animal Studies

The role of dendritic cells in DKD is supported by findings from a mouse model of DKD, in which dendritic cells were observed within the glomerulus in close proximity to T- and B-cells. These immune infiltrates were associated with glomerular expansion and albuminuria [36]. Further evidence of the interaction between dendritic cells and cells from the adaptive immune system is seen in a rat model of DKD, where CD11c^+^ CD103^+^ dendritic cells were significantly increased and contributed to DKD by activating CD8^+^ T-cells [37]. Mesenchymal stem cell transplantation attenuated renal fibrosis and CD103^+^ dendritic cell and CD8^+^ T-cell infiltration, suggesting they play a pathogenic role. Although dendritic cells may play a role in DKD by activating innate immunity in animal models, it remains to be determined whether dendritic cells play a pathogenic role in DKD in humans.

## 3. Adaptive Immunity

### 3.1. T-Cells

The innate immune system is important for activating the adaptive immune response as antigen-presenting cells. Dendritic cells are activated in response to external stimuli such as infection, leading to dendritic cell antigen presentation to T-cells, with further T-cell activation by costimulatory molecules and cytokines [38]. The adaptive immune system also plays a role in the pathogenesis of DKD. Conventional T-cells include CD4^+^ and CD8^+^ T-cell subsets, with several CD4^+^ T-cell subtypes including T helper cells or T regulatory cells [39]. There are also resident memory T-cells in the kidney [40]. T-cells are capable of secreting cytokines such as IL-2, IL-17, TNF-α, and interferon-γ (IFN-γ), which are increased in the serum of people with T2D and DKD [41]. T-cell recruitment has also been shown to be an important process in DKD. It has been hypothesized that release of cytokines and chemokines amplifies T-cell recruitment in DKD, though the exact mechanism by which this process occurs is not well elucidated [40].

Animal Studies

The pathogenic role of T-cells in DKD is supported by studies in animal models. In a Rag^−/−^ mouse model of T1D deficient in T- and B-cells, investigators found reduced albuminuria and podocyte loss; however, they still exhibited macrophage accumulation and histological features of kidney disease due to diabetes [42]. This study suggests that although adaptive immunity plays a role in contributing to albuminuria, macrophages still play a significant role in tubulointerstitial injury and progressive kidney function decline.

T-cells may also play a direct effect on recruiting macrophages to the kidney via cytokine secretion. This is observed in STZ-mice which had an increased number of CD4^+^ and CD8^+^ T-cells in the kidneys compared to controls. Importantly, there was a concordant increase in IFN-γ and TNF-α mRNA expression in the kidney and higher secretion of both cytokines from T-cells [43]. This is of relevance, as IFN-γ and TNF-α are hypothesized to recruit macrophages to the kidneys, thereby demonstrating a direct role of T-cells in propagating macrophage infiltration. Conversely, renal tubular cells also play a role in the recruitment of T-cells to the kidney. ScRNA-seq data from a mouse model of T2D DN revealed higher CXC chemokine ligand 12 (CXCL12) expression in the proximal tubules compared to controls, which was accompanied by T-cell infiltration [44]. In this model, treatment with CXCL12 antibodies attenuated effector T-cell infiltration in the kidney, suggesting a role of CXCL12 in the recruitment of T-cells [44].

Human Studies

The importance of T-cells in DKD was documented in several human studies. Studies of individuals with T1D showed T-cell infiltration in the juxtaglomerular apparatus in a subset of people with T1D [45]. Additional evidence of the pathogenic role of T-cells in humans is seen in a study of people with confirmed DN on kidney biopsy. Those with higher CD4^+^ T-cell infiltration had more severe glomerular lesions, tubular atrophy, interstitial fibrosis, lower eGFR, and higher proteinuria [46]. Several potential mechanisms have been found to explain T-cell mediated injury in DKD. Kidney injury molecule-1 (KIM-1) is a transmembrane receptor on T-cells that leads to T-cell activation and cytokine production to induce proximal tubular injury [47]. People with T1D at high DKD risk had higher plasma T-cell KIM-1 expression and KIM-1 T-cell infiltration in the kidney [47]. Plasma KIM-1 levels were positively correlated with albuminuria. This suggests that KIM-1 expressing activated T-cells may play a role in the pathogenesis of DKD in humans. Another potential mechanism by which T-cells can mediate kidney injury is via T-cell ligands, IL-17A and CD40, both of which stimulate cytokine release from antigen-presenting cells and kidney epithelial cells, respectively [48]. T-cell products IL-17A and CD40 ligand were observed in kidney biopsies of people with T2D. To further identify their role, in vitro studies showed that IL-17A and CD40 ligand synergistically stimulated podocyte secretion of IL-6, MCP-1, RANTES, NF-κB, and TGF-β1 to further enhance leukocyte recruitment [48].

### 3.2. B-Cells

There are limited studies on the role of B-cells in DKD. B-cells can potentially contribute to the pathogenesis of DKD by losing anergy and becoming activated after interacting with T-cells, inducing cytokine secretion [49].

Animal Studies

The presence of B-cells was observed in a mouse model, which exhibited infiltration of T-cells, IgG^+^ B-cells, and dendritic cells in early stages of DKD [36].

Human Studies

People with DN had higher number of CD38^+^CD19^+^ and CD38^+^CD19^+^CD40^+^ B-cells in the blood, as well as IL-21, compared to healthy controls without CKD [50]. This finding is further supported by a study in which scRNA-seq from kidney biopsy tissues from individuals with DKD revealed a 7- to 8-fold increase in leukocyte infiltration compared to nondiabetic controls with CKD, with B-cells comprising 21% of the infiltrating immune cells [51]. Furthermore, scRNA-seq data from glomerular cells of people with DKD revealed a higher abundance of B-cells compared to nondiabetic controls without CKD [52]. In addition, these B-cells also exhibit altered function in people with DKD. In the same analysis, B-cells of individuals with DKD had reduced A disintegrin and metalloproteinase 28 gene expression, which are involved in B-cell proliferation, suggesting the presence of B-cell dysfunction in people with DKD [52]. Altered B-cell tolerance in people with DKD adds to the evidence for B-cell dysfunction in DKD. Regulatory B-cells secrete IL-10, which play a role in inhibiting inflammatory responses mediated by T-cells. People with DKD had lower CD19^+^CD24^hi^CD38^hi^ regulatory B-cells than both diabetic and nondiabetic controls without CKD. This decline in regulatory B-cells positively correlated with eGFR decline and serum IL-10, and negatively correlated with urinary protein levels [53]. Thus, it is hypothesized that a decrease in regulatory B-cells allows for enhanced T-cell activation through reduced regulatory cytokines, including IL-10.

## 4. Soluble Mediators

### 4.1. Chemokines

Chemokines play an important role in the recruitment of monocytes to the kidney in DKD.

#### 4.1.1. CCR2

The MCP-1/CCR2 axis has been reported as a mediator of monocyte recruitment in DKD. CCR2 is present on monocytes/macrophages, T-cells, and dendritic cells, and binds to MCP-1. In the setting of DKD, kidney parenchymal cells including podocytes, tubular, and mesangial cells secrete MCP-1, which binds to CCR2 on circulating monocytes to stimulate their activation and recruitment into the kidney, leading to kidney injury [54].

Animal Studies

The importance of MCP-1 in DKD is seen in a mouse model of T1D, where MCP-1 deficient mice had reduced glomerular and tubulointerstitial macrophage infiltration, fibrosis, albuminuria, and creatinine levels compared to MCP^+/+^ mice [55]. This suggests that MCP-1 may play a role in recruiting monocytes to contribute to DKD. In vitro studies showed CCR2 is also present in T-cells, and it is hypothesized that MCP-1 plays a role in T-cell recruitment to sites of inflammation [40,56]. However, a MCP-1 deficient diabetic mouse model of DKD had no impact in CD4^+^ and CD8^+^ T-cell recruitment into the kidney [55]. Therefore, it is possible additional mechanisms are also at play, and the exact role of MCP-1 in the recruitment of T-cells in DKD needs to be further elucidated.

Human Studies

In humans, the importance of the MCP-1/CCR2 axis was studied in a double-blind, placebo-controlled trial involving 332 patients with T2D and albuminuria. Treatment with CCX140-B, a CCR2 inhibitor for 52 weeks, in addition to standard of care, reduced albuminuria by 18% compared to placebo (2%). This underscores the importance of attenuating inflammatory monocyte recruitment in DKD [57]. However, given the relatively modest effect of CCR2 blockade, it is likely that the pathogenesis of monocyte recruitment is more complex, and further studies are required to elucidate additional pathways involved in monocyte recruitment into the kidneys and DKD in humans.

#### 4.1.2. CX3CR1

C-X3-C motif chemokine ligand 1 (CX3CL1) is a chemokine whose expression is increased in the kidney in response to injury. CX3CL1 facilitates monocyte recruitment by binding to the C-X3-C motif chemokine receptor 1 (CX3CR1) on monocytes [58]. CX3CR1 is expressed on monocyte/macrophages, T-cells, dendritic cells, and mast cells [58].

Animal Studies

The importance of the CX3CL1-CX3CR1 axis was demonstrated in a rat model of diabetes, where CX3CL1 mRNA expression was upregulated with a resultant increase in kidney infiltrating cells expressing CX3CR1, including macrophages [59].

Human Studies

The animal model findings described above were confirmed in humans with DKD, where CX3CR1 was upregulated in people with diabetic tubulopathy [60].

#### 4.1.3. CCL5

C–C chemokine ligand 5 (CCL5) is a chemokine that binds to the C–C chemokine receptor 5 (CCR5) expressed on monocyte/macrophages, T-cells, and dendritic cells [61].

Human Studies

The CCR5 promoter 59029 A genotype was associated with albuminuria in people with T2D compared to people with T2D and normoalbuminuria [62]. Given the potential role of the CCL5 axis in DKD, the efficacy of dual CCR2/CCL5 blockade was tested in a phase 2 randomized, double-blind, placebo-controlled study of people with T2D and CKD. Those in the treatment arm experienced an 8.2% reduction in the albuminuria after 12 weeks of therapy, compared to the placebo arm. Due to its modest effect in humans, more effective treatment options need to be considered [63].

### 4.2. Adhesion Molecules

#### 4.2.1. ICAM-1

Another important molecule in the recruitment of monocytes is ICAM-1. ICAM-1 plays an important role in the recruitment of leukocytes to the kidney by facilitating their binding to endothelial and epithelial cells, and its expression can be stimulated by cytokines in the diabetic milieu [64,65].

Animal Studies

In a STZ rat model, there was higher ICAM-1 expression in the glomerulus and interstitium, with a resultant increase in kidney infiltration by monocytes/macrophages and lymphocytes [66]. In addition, blocking ICAM-1 with a monoclonal ICAM-1 antibody prevented mononuclear infiltration in this model [66]. ICAM-1 may also be important in the recruitment of T-cells in DKD. In db/db mice, the increase in glomerular CD4^+^ T-cells was absent in animals treated with a monoclonal ICAM-1 antibody, suggesting a potential role for ICAM-1 in T-cell recruitment in DKD [65].

Human Studies

The importance of ICAM-1 was also observed in individuals with T1D and DKD who were part of the Diabetes Control and Complication Trial and the Epidemiology and Diabetes and Complications study, where high serum levels of soluble ICAM-1 were associated with a higher relative risk of incident sustained microalbuminuria over time [67].

#### 4.2.2. VCAM-1

VCAM-1 is another adhesion molecule expressed in the kidneys that facilitate leukocyte (mononuclear and T-cell) recruitment [68]. VCAM-1 gene expression can be stimulated in response to cytokine release, such as TNF-α or IL-1 [69].

Animal Studies

In diabetic KKAy mice, VCAM-1 expression was observed in the kidney vascular endothelium and interstitium, in addition to lymphocytes, and monocytes, when compared to nondiabetic controls without CKD [70].

Human Studies

Plasma soluble VCAM-1 and ICAM-1 levels were elevated in individuals with T1D with microalbuminuria and proteinuria compared to healthy controls without diabetes or CKD [71]. These studies suggest ICAM-1 and VCAM-1 expression in the kidneys may play a role in DKD by recruiting monocytes and T-cells, which induces kidney injury.

### 4.3. Cytokines

Several cytokines have been found to be significant in DKD, including TNF-α, IL-6, and IL-1.

#### 4.3.1. TNF-α

TNF-α is secreted by leukocytes such as mononuclear and T-cells, but can also be secreted by intrinsic kidney cells such as podocytes, mesangial, endothelial, and tubular cells [72]. TNF-α and TNF receptor (TNFR) 1 and 2 are proteolytically cleaved by A disintegrin and metalloproteinase 17 at the cell surface to release the soluble forms of TNF-α and TNFR 1 and 2 [73]. TNF-α can bind to TNFR1 or 2 to induce specific signaling changes in the cell. It is believed that TNF-α is a key propagator of injury in DKD upon release from infiltrating monocytes by inducing further cytokine, adhesion molecule or chemokine release, and by exerting direct effects in the kidney [72].

Animal Models

The direct cytotoxic effects of TNF-α on podocyte apoptosis were observed in a previously mentioned in vitro study in which podocytes exposure to high glucose, and co-culture with murine macrophages had higher apoptosis, which was mediated by TNF-α [20]. TNF-α was also shown to induce injury to several other kidney cell types in vivo. Further implicating TNF-α, a study of STZ-diabetic rats treated with TNF-α blockade showed reduced albuminuria, glomerular, and tubular injuries due to reductions in NOD-like receptor family pyrin domain containing 3 inflammasome activation and oxidative stress [74]. Moreover, Ins2^Akita^ mice treated with a TNF-α blocking antibody had reduced macrophage infiltration and histologic lesion in their kidney, exhibiting reductions in albuminuria and serum creatinine compared to vehicle-treated mice [75]. These animal model studies indicate that TNF-α may play a role in kidney disease due to diabetes by both exerting direct cytotoxic effects on the kidney and indirectly by recruiting immune cells.

Human Studies

The importance of TNF-α was also demonstrated in humans with DKD. In people with T2D and DKD, albuminuria was positively correlated with both serum and urinary TNF-α levels [76]. Additionally, in the Joslin Study of the Genetics of Type 2 Diabetes and Kidney Complications, plasma circulating TNFR1 and 2 predicted the risk for end-stage kidney disease [77]. These studies provide support for an association between TNF-α and clinical outcomes of DKD in humans.

#### 4.3.2. IL-6

IL-6 is secreted by mononuclear cells, T-cells, and mesangial, glomerular epithelial, and tubular cells of the kidney.

Animal Studies

In a STZ diabetes rat model, IL-6 levels were elevated in the serum, urine, and renal cortex compared to nondiabetic rats. Renal hypertrophy also correlated with both renal and urinary IL-6 levels, suggesting a potential role for IL-6 in the early pathological changes of DKD [78]. Additionally, blocking IL-6 with Tocilizumab in db/db mice attenuated the histological changes of DKD, proteinuria, and insulin resistance, to suggest a potential pathogenic role [79].

Human Studies

In humans with T2D, serum levels of IL-6 were positively correlated with the degree of glomerular basement membrane thickening [80].

#### 4.3.3. IL-1

IL-1 is secreted by mononuclear cells, and intrinsic cells of the kidney. IL-1 contributes to the pathological changes in DKD by inducing mesangial expansion and adhesion molecular expression in the glomerulus and tubules [81].

Animal Studies

In a STZ rat model, there was higher expression of IL-1 in the kidney cortex compared to nondiabetic controls [78].

Human Studies

In individuals with T2D and DKD, elevated serum IL-1α levels correlated with markers of podocyte and proximal tubular injury, further supporting a role for IL-1α in disease progression [82].

## 5. Translational/Therapeutic Potential

With improved understanding of the complex role of the innate and adaptive immune systems in DKD, new potential therapeutic targets have emerged. Both CCL2 and CCR2 inhibition were investigated in people with DKD. In a randomized, double-blind, placebo-controlled phase II study of 75 people with T2D and albuminuria, CCL2 inhibition reduced urinary albumin/creatinine ratio after 3 months by 29% from baseline (*p* < 0.05) compared to 15% reduction in the placebo group (*p* = 0.221) [83]. As previously mentioned, CCR2 inhibition in 332 patients with T2D and albuminuria for 52 weeks also reduced albuminuria by 18% compared to placebo (2% reduction) [57]. Another area of investigation is direct cytokine inhibition. Pentoxifylline, a xanthine derivative, significantly reduced TNF-α in people with CKD without diabetes compared to usual care after 12 months [84]. Those in the pentoxifylline group did not experience changes in urinary albumin excretion or eGFR compared to a decline in eGFR in the usual care group (40.1 ± 12.4 to 35.7 ± 13.4 mL/min/1.73 m^2^, *p* < 0.001) [84]. Newer agents have emerging evidence in mediating inflammation in DKD. Our prior work showed that high glucose in vitro stimulated TNF-α secretion from monocytes of healthy people, which induced podocyte apoptosis. Treatment of monocytes with a GLP-1 RA attenuated monocyte TNF-α secretion, thereby reducing podocyte apoptosis [22]. Treatment of people with T2D with empagliflozin, a SGLT2i, for 30 days reduced macrophage secretion of IL-1β compared to people treated with a sulfonylurea [85]. Finally, people with T2D treated with finerenone, a non-steroidal MRA, for 6 months had reduction in albuminuria and IL-1β, TNF-α, and IFN-γ compared to baseline [86]. Taken together, early human studies from GLP-1 RA, SGLT2i, and finerenone may show an anti-inflammatory effect of these medications via modulating activation of monocytes/macrophages. Despite these promising results, more effective approaches are needed to identify agents with greater efficacy and specificity in reducing kidney disease progression by inhibiting immune-mediated injury.

## 6. Conclusions

Diabetes is a leading cause of end-stage renal disease and has been linked to increased morbidity and mortality. Growing evidence supports a critical role of inflammation in the development and progression of DKD. However, much of the existing data are derived from animal models, and the key mechanisms driving immune activation and kidney injury in humans with DKD remain unclear. Despite the complexity of immune activation in DKD, several common mediators are important including cytokines which are secreted from both intrinsic kidney cells and immune cells. Our review highlights the bidirectional crosstalk between monocyte/macrophages and intrinsic kidney cells. Remaining questions for future research include the exact role of immune cells in nonalbuminuric form of DKD in humans. Additionally, the role of neutrophils and dendritic cells in DKD, and the mechanisms which drive interactions between the innate immune and adaptive immune system in humans, remain to be clarified. A more complete understanding of the key mechanisms regulating immune activation in people with DKD will allow for the development of newer therapies to effectively target immune activation in DKD.

## Data Availability

No new data were created or analyzed in this study. Data sharing is not applicable to this article.
